# Treating Symptomatic Aortic Stenosis With Transcatheter Aortic Valve Replacement: Is There Time to Wait?

**DOI:** 10.1161/JAHA.118.011527

**Published:** 2019-01-05

**Authors:** Kamil F. Faridi, Robert W. Yeh, Marie‐France Poulin

**Affiliations:** ^1^ Richard A. and Susan F. Smith Center for Outcomes Research in Cardiology Department of Medicine Beth Israel Deaconess Medical Center Harvard Medical School Boston MA; ^2^ Division of Cardiology Department of Medicine Rush University Medical Center Chicago IL

**Keywords:** Editorials, aortic stenosis, aortic valve replacement, quality of care, transcatheter aortic valve replacement, wait times, Aortic Valve Replacement/Transcather Aortic Valve Implantation, Catheter-Based Coronary and Valvular Interventions, Health Services, Quality and Outcomes

## Introduction

The transcatheter aortic valve replacement (TAVR) landscape in the United States has significantly changed over the past 5 years. Not only has the technology improved, but with the approval of TAVR for patients at intermediate risk for surgical aortic valve replacement, the number of patients who are eligible for a TAVR procedure has increased considerably. In 2012, there were 198 sites in the United States performing >4600 TAVR procedures. By 2018, the number of sites had climbed to 580, with >100 000 TAVR procedures performed during the intervening years.^1^, ^2^ In 2015, the annual number of TAVR procedures surpassed the number of surgical aortic valve replacements for the first time.^3^ Worldwide, TAVR for treatment of aortic stenosis has an estimated growth of 40% per year.^4^ Results of clinical trials further investigating the role of TAVR in low‐risk patients and those with asymptomatic severe aortic stenosis are also expected in the coming years.^5^ As the number of patients meeting the expanding indications for TAVR continues to grow, the impact of potentially longer wait times may become increasingly important.

There is currently no consensus on what an acceptable wait time before TAVR should be. Recent multisociety consensus documents do not address this issue.^2^, ^6^ Some have suggested a maximum wait of 60 days, although this threshold has not been clinically validated.^7^ In Canada, a reported median time from referral to TAVR procedure using observational data was 80 days.^8^ Despite the large number of TAVR centers in the United States, centers are significantly heterogeneous in terms of procedural volume and expertise.^2^ Current wait times for existing programs across the country remain unknown. Factors influencing wait time include availability of local expertise and capacity of medical centers to take increasing volumes of patients. Inefficient coordination of care may also lead to longer wait times, given that TAVR workup requires multiple different outpatient evaluations and testing. To better understand the causes of delay, if any, and the impact of TAVR wait time on outcomes in the United States, it would be highly desirable to have information on wait times and their causes captured in TAVR registries moving forward.

In this issue of the *Journal of the American Heart Association (JAHA)*, Wijeysundera and colleagues investigated the impact of wait times on outcomes at 30 days following TAVR.^9^ They conducted a retrospective analysis of 2170 patients who received a TAVR procedure between 2010 and 2016 using the TAVR CorHealth Registry data from 10 hospitals in Ontario, Canada. They evaluated whether wait time was associated with increased mortality or hospital readmission within 30 days following a TAVR procedure. Patients who were hospitalized and subsequently underwent TAVR during that admission were considered to have undergone an urgent procedure.

In the study, the median time from referral to TAVR procedure was 107 days with an interquartile range of 55 to 176 days. Patients who underwent elective TAVR (80.2% of the cohort) had a median wait time of 124 days (interquartile range: 72–189 days), whereas those who underwent urgent TAVR (19.8%) had a median wait time of 36 days (interquartile range: 14–95 days). Contrary to the authors’ hypothesis, shorter wait times were significantly associated with increased mortality (*P*<0.001) and 30‐day readmission (*P*=0.01) in unadjusted models; significant associations remained after adjusting for clinical variables. However, they found that when urgency status of TAVR was included in the multivariate model, there was no longer a significant association between wait times and mortality (*P*=0.58) or 30‐day readmission (*P*=0.98). When patients who underwent urgent and elective TAVR were analyzed as separate groups, the authors again found no associations between wait times and outcomes. The authors concluded that the relationship between wait times and postprocedural outcomes was mediated entirely by urgency status.

Patients who received an urgent TAVR were sicker, with higher baseline rates of heart failure, renal disease, and arrhythmia. They also had longer TAVR hospitalizations (mean: 15.1 versus 8.6 days for elective patients). Consequently, it should come as no surprise that urgent TAVR patients also had worse unadjusted post‐TAVR mortality compared with elective procedures (11.4% versus 5.7%; *P*<0.001) and 30‐day readmission (20.3% versus 14.5%; *P* value 0.003), along with higher rates of procedural complications including acute kidney injury and bleeding.

Although Wijeysundera and colleagues suggest that the association between short wait time and worse post‐TAVR mortality without accounting for urgency status is paradoxical, this is actually what can be expected from clinical practice. Short TAVR wait time is not a random event but rather a result of treatment decisions based on clinical status. Patients who are too unstable to be discharged and are scheduled for an elective TAVR (therefore requiring an urgent TAVR) are, by definition, sicker and more likely to have worse outcomes than patients who can be stabilized and discharged home, in both measured and unmeasured ways. This makes studying the true independent effect of TAVR wait time using observational data very challenging. Given the clear concern of confounding by indication, urgency status of the procedure needs to be accounted for when studying the impact of wait times for TAVR. The authors recognized this issue and addressed it by correcting for the urgency status of the procedure in their analyses.

However, even after stratifying by urgency status, there still may be clinical factors that drive wait times and could confound the study. Although this study found no significant association between wait times and adverse outcomes following TAVR, these results should nevertheless be interpreted with caution. The primary end points were post‐TAVR outcomes, and the analyses do not fully account for what happened to patients while waiting. In this study, the rate of all‐cause hospitalization after being placed on the wait list was nearly 40%, excluding the index admission of patients who required an urgent TAVR, with 9.1% of hospitalizations being for acute heart failure. It is also important to note that patients who died while waiting for TAVR (suggested in this study to be 5% of the pre‐TAVR population) were excluded from the analysis. Mortality has ranged from 2.0% to 14% in other studies while waiting for a TAVR, which, again, is not negligible and is a potential complication of longer wait times.^8^, ^10^, ^11^, ^12^ Longer wait times before an aortic valve replacement (TAVR or surgical aortic valve replacement) have also been associated with decline in functional status and worse prognosis.^10^, ^13^, ^14^, ^15^ Similarly, patients with severe symptomatic aortic stenosis who previously declined TAVR but subsequently changed their minds and underwent a TAVR months later have significantly higher 30‐day and cumulative 1‐year mortality than other patients.^16^ The majority (75%) changed their minds after an acute heart failure episode. This brings up the importance of patient selection for TAVR. “Cohort C” patients, whose comorbidities and poor functional status would negate any potential benefits of a TAVR procedure, are also more likely to die before and after their procedure. Until more definitive data are available, centers should focus on careful patient selection and continued prompt scheduling of preprocedural testing and the TAVR procedure.

The authors also call for better ways to identify patients who may deteriorate and require unplanned hospitalization and urgent TAVR. Risk scores accounting for various patient characteristics are needed to identify patients at high risk of requiring an urgent TAVR. Implementation of such prediction models would help TAVR centers triage such patients and “fast track” their care while patients are still clinically stable and medically optimized. Comorbidities such as prior heart failure and advanced renal failure, for example, may be useful predictors. Other variables not measured in data from this study, such as symptom severity, severity of aortic stenosis, left ventricular ejection fraction, and New York Heart Association classification, may also prove to be useful predictors. Further investigation is needed to determine which characteristics may be most useful for risk prediction when initially evaluating TAVR candidates.

An additional factor to consider is the timing of initial TAVR referral. This is typically dependent on primary care providers or general cardiologists, who should be aware of current indications for valve replacement and refer to proceduralists at the appropriate time. It has long been recognized that patients with severe aortic stenosis should undergo valve replacement once symptoms develop. However, up to 50% of patients with severe aortic stenosis may be inaccurately perceived as being asymptomatic at time of diagnosis. Low‐intensity exercise stress testing may unmask symptoms in some sedentary patients. Development of symptoms, decreased exercise tolerance, or a fall in blood pressure while exercising are all indications to consider valve replacement.^17^ Certain groups of asymptomatic patients warrant consideration for valve replacement because of high risk of disease progression and adverse outcomes. These include patients with severe aortic stenosis and left ventricular ejection fraction ≤50%, extremely severe stenosis (peak transvalvular velocity ≥5.0 m/s or mean gradient ≥60 mm Hg), peak transvalvular velocity progression ≥0.3 m/s per year, markedly elevated natriuretic peptide on repeated testing, and excessive left ventricular hypertrophy in the absence of hypertension.^18^

In summary, Wijeysundera and colleagues should be commended for tackling this intriguing clinical question in patients waiting for a TAVR procedure. Although the impact of wait time on postprocedure outcomes seems to be driven primarily by clinical status based on these data, big questions remain. As healthcare workers, it is clear that we need to continue researching and developing tools and systems that will optimize outcomes in the pre‐TAVR population.

## Disclosures

Dr Yeh is a consultant and a member of scientific advisory boards for Abbott Vascular, Boston Scientific, and Medtronic and has received investigator‐initiated research grants from Abbott Vascular and Boston Scientific. The remaining authors have no disclosures to report.
